# Ownership and utilization of mosquito bed net among pregnant women in Ghana: a national population-based survey

**DOI:** 10.1186/s41182-025-00739-z

**Published:** 2025-05-09

**Authors:** Wise Awunyo, David Gameli Agbleta, Mary Adaeze Udeoha, Matilda Mawusi Kodjo, Agani Afaya

**Affiliations:** Department of Nursing, School of Nursing and Midwifery, University of Health and Allied Sciences, Ho, Ghana

**Keywords:** Malaria, Pregnancy, Mosquito bed nets, Ownership, Utilization, Ghana Demographic and Health Survey

## Abstract

**Background:**

Despite mosquito bed net use being among the many evidence-based safe and successful interventions to avert plasmodium infection during pregnancy, its use remains low among pregnant women due to various barriers. This study, therefore, examined the factors associated with mosquito bed net utilization and ownership among pregnant women in Ghana.

**Methods:**

Data from the 2022 Ghana Demographic and Health Survey were analyzed for this cross-sectional study. A representative sample of 1111 pregnant women from the 16 regions of Ghana were included in the study. Multivariate logistic regression analysis was used to determine the factors associated with mosquito bed net ownership and utilization among pregnant women of reproductive age.

**Results:**

The prevalence of mosquito bed net ownership among pregnant women was 80.1% [CI 76.8–83.0] and that of mosquito bed net utilization was 47.6% [CI 43.9–51.4]. We also found that pregnant women with 1–3 children [aOR = 2.07, 95% CI 1.25–3.43] and 4 or more children [aOR = 2.52, 95% CI 1.38–4.59], had a partner with secondary level education [aOR = 2.11, 95% CI 1.10–4.06] and higher educational status [aOR = 2.47, 95% CI 1.06–5.74] had higher odds of mosquito bed net use. However, pregnant women who belonged to middle wealth quintiles [aOR = 0.41, 95% CI 0.21–0.82], richer wealth quintiles [aOR = 0.19, 95% CI 0.09–0.41], and richest wealth quintiles [aOR = 0.09, 95% CI 0.03–0.25] had decreased odds of mosquito bed net use. In addition, pregnant women who had female household heads [aOR = 1.73, 95% CI 1.03–2.91], resided in the rural areas [aOR = 1.97, 95% CI 1.12–3.49], had 1–3 children [aOR = 1.65, 95% CI 1.05–2.58] and 4 or more children [aOR = 2.08, 95% CI 1.03–4.20] had higher odds of mosquito bed net ownership. Meanwhile, pregnant women with active health insurance [aOR = 0.29, 95% CI 0.10–0.80] had lesser odds of mosquito bed net ownership.

**Conclusion:**

The rate of mosquito bed net ownership was high among pregnant women. However, the effective utilization of mosquito bed net among pregnant women was low. Considering the low utilization of mosquito bed nets, public health practitioners and clinicians should develop awareness and educational interventions tailored toward improving mosquito bed net use among pregnant women.

## Background

Pregnant women and children under 5 years are disproportionately affected by malaria, a disease caused by mosquito bites spread by female Anopheles mosquitoes that continues to be a major global health and socioeconomic concern [1, 2]. An estimated 263 million cases of malaria were reported in 2023, with 597,000 fatalities, an increase of 11 million cases over 2022 [1, 3]. Incidence rates have not changed over the past 3 years, after rising by 3% in 2020. In 2022, there were 58 cases of malaria for every 1000 people at risk. With an estimated 233 million cases, the African Region accounted for nearly 94% of all cases worldwide [1]. Low- and middle-income countries (LMICs) accounted for over 96% of malaria deaths globally [1, 2]. Nigeria (31%), the Democratic Republic of the Congo (12%), Niger (6%), and the United Republic of Tanzania (4%) were responsible for slightly more than half of all malaria deaths in 2022 [1]. In the WHO African Region, malaria mortality decreased from 808,000 in 2000 to 548,000 in 2017 before increasing to 604,000 in 2020 [1, 2, 4]. Between 2000 and 2019, the malaria mortality rate decreased by 60%, from 143 to 57 deaths per 100,000 at-risk individuals. It subsequently increased to 61 in 2020 and then dropped to 56 in 2022 [1].

Malaria is the leading cause of illness and death in Ghana, particularly among children under the age of five and pregnant women [2].

Ghana's malaria burden remains significant, with malaria being one of the top three illnesses encountered in outpatient clinics [2]. Ghana accounts for 2.2% of malaria cases and fatalities worldwide and 4% of cases in West Africa, making it one of the 15 nations with the greatest malaria burden [5, 6]. In 2022, the government documented around 5.2 million verified malaria infections, and 151 fatalities related to malaria. In the same year, the in-patient malaria mortality rate was 0.48 per 100,000 persons, compared to 0.05 per 100,000 for children under the age of five. Malaria transmission stays steady in Ghana, with varying endemicity among areas [2]. According to the Ghana Demographic and Health Survey (2022), the nationwide prevalence fell from 14.1% in 2019 to 8.6% in 2022 [7]. The Greater Accra Region continues to have the lowest prevalence of 2.0%, with the Oti Region having the highest at 15.0% [2]. Malaria cases among pregnant women in Ghana increased from 383,034 in 2016 to 399,736 in 2017 [8]. The number of cases among pregnant women increased by 4.2% compared to 2016. Malaria in pregnancy [MIP] poses significant dangers for women and unborn children by raising maternal anemia and unfavorable birth outcomes, such as low birth weight, premature birth, stillbirth, and maternal and infant death. To assist in decreasing Malaria in Pregnancy (MIP), the National Malaria Control Program (NMCP) has implemented a range of preventable measures, such as Intermittent Preventive Treatment of Malaria in Pregnancy (IPTp), the supply and utilization of mosquito bed nets, and Indoor Residual Spraying [7, 9].

Using a mosquito bed net is one of the best and most effective strategies to avoid malaria infection during pregnancy [1]. Mosquito bed nets are a key component of prevention efforts, which are essential to lowering the malaria burden. Mosquito bed nets are extensively acknowledged for their ability to physically prevent mosquito bites, particularly during peak biting hours, hence limiting the spread of malaria [10]. Using a variety of channels, including recurring public campaigns, school-based distribution, and continuous programs targeted at expectant mothers and children, the distribution of mosquito bed nets is an essential part of Ghana's malaria prevention efforts [10]. To ensure malaria is prevented, mosquito bed nets are administered during antenatal care, which have also been shown to lower the overall fatalities by 17% worldwide and malaria episodes in children under five by almost 50% [1, 7, 11]. According to studies, pregnant women and children under the age of five in SSA rarely utilized mosquito bed nets [12]. Poor mosquito bed net utilization is caused by a combination of sociodemographic factors, a lack of knowledge and awareness, and mosquito bed net-related concerns (such as availability, adequacy, quality, physical state of maintenance repair, and effectiveness) [13–16]. Malaria is still prevalent among pregnant women in sub-Saharan African nations, causing considerable morbidity and death despite the availability of effective preventative methods [1]. Malaria interventions among pregnant women, such as rapid use of mosquito bed nets and IPTp, have not always been successful, highlighting ongoing challenges that must be addressed.

The WHO recommended using mosquito bed nets as an evidence-based intervention to help reduce the prevalence of malaria. The implementation of prenatal care services in Ghana made it possible to distribute mosquito nets and educate pregnant women about malaria prevention. However, despite various targeted interventions implemented in Ghana to increase the acquisition of mosquito bed nets and public knowledge of malaria, as well as its roles in reducing the prevalence and impact of malaria among pregnant women, the usage of mosquito bed nets remains low among pregnant women. This may be as a result of unwillingness to register or redeem mosquito bed nets, as well as a preference for other mosquito control approaches [10]. Also, there have been some cultural beliefs that the mosquito bed nets are unclean [17]. Aside from the cultural beliefs, logistical challenges, such as staffing and short periods of campaigns, as well as the perceived discomfort of sleeping beneath nets [18, 19] impact the use of mosquito bed nets. Environmental factors, such as warm and humid temperatures, the scent of the net, and the problems associated with hanging the net, also inhibit the use of mosquito bed nets regularly, since people may find them unpleasant when sleeping [20] (climate change, which includes shifting rainfall patterns, temperature changes, humidity, deforestation, and other forms of environmental degradation) has been documented in Ghana. These changes may influence malaria epidemiology and the implementation of malaria prevention and control programs.

This, however, leads to no evidence of a decrease in the prevalence and burden of malaria among pregnant women, as malaria remains one of the top causes of morbidity and mortality among pregnant women in Ghana. The purpose of this study was to assess the factors associated with pregnant women's ownership and use of mosquito bed nets across the 16 regions in Ghana. The study's findings will provide policymakers, implementers, and researchers with up-to-date information on the proportion of pregnant women in Ghana who own and use mosquito bed nets, as well as the factors that influence ownership and utilization of mosquito bed nets.

## Methods

### Data source and setting

This cross-sectional survey was conducted among the 16 regions in Ghana. This study used data from the 2022 Ghana Demographic and Health Survey (GDHS), which was implemented by the Ghana Statistical Service (GSS). Data were collected between October 17, 2022, and January 14, 2023. GSS received technical assistance from the International Coaching Federation (ICF) for the Demographic and Health Survey Program (DHS) to make sure the survey methodologies adhered to ethical research guidelines. Several agencies and organizations funded the survey, including the Government of Ghana, UNFPA, UNICEF, the World Bank, the Global Fund, the Korean International Cooperation Agency (KOICA), the WHO, and the Foreign, Commonwealth, and Development Office (UK AID), to ensure its successful implementation. The 2022 GDHS collected data on demographic and health factors, including age, educational status, household wealth, and ownership and use of mosquito nets. According to the 2021 Population and Housing Census, Ghana has a total population of 30.8 million people, with 15.6 million women and 15.2 million males living in 16 regions. It shares borders with Burkina Faso to the north, Togo to the east, Cote d'Ivoire to the west, and the Gulf of Guinea to the south [7]. Accra is the capital city. The study was conducted and relied on strengthening the Reporting of Observational Studies in Epidemiology (STROBE) guidelines.

### Population and sampling

Ghana Statistical Service modified the sampling frame for the 2022 GDHS using data from the 2021 Population and Housing Census. The sampling approach used in the 2022 GDHS was stratified two-stage cluster sampling, which was designed to generate representative results at the national level, for urban and rural areas, and for each of the 16 regions. During the first step, 618 target clusters were selected using a probability proportional to size (PPS) technique for urban and rural areas within each region. The target number of clusters was then picked with equal probability by systematic random selection of the clusters chosen in the first phase, for both urban and rural areas in each region. The second stage involved listing and updating the households in each cluster selected to create a list. This list was used to choose a sample of households. Prior to the listing and mapping, GSS arranged a 5-day listing process training program for listers and mappers, with ICF help. The listers captured geographical coordinates for each dwelling using GPS dongles provided by ICF, in accordance with the DHS Listing Manual instructions. The DHS Program provided software for home listing, which was completed using tablet computers. Interviews were carried out with 30 randomly selected houses from each cluster. For the 2022 GDHS, a national stratified representative sample of 18,450 households was selected from 618 clusters. GSS conducted a pre-test of the survey questionnaire and provided staff training before collecting data. Women aged 15–49 were recognized as eligible survey participants. A total of 15,014 women of reproductive age were interviewed. This study analyzed data from 1111 pregnant women (weighted) from the 16 regions in Ghana. Understanding the preventative behaviors of pregnant women of reproductive age (15–49 years) is critical for reducing malaria and preventing mortality. Prior to the survey, all eligible participants provided verbal informed consent, which was noted on the survey questionnaire itself.

### Outcome variables

The outcome variables assessed whether pregnant women own and use mosquito bed net for sleeping. Participants who were eligible for the study were asked whether they slept under a mosquito bed net and the response was coded “0’ for “No” indicating pregnant women who did not sleep under a mosquito bed net and “1’ for “Yes” representing those who confirmed sleeping under a mosquito bed net. They were also asked whether respondents have mosquito bed net for sleeping. We coded “0” for “No” (pregnant women who do not have mosquito bed net) and “1” for “Yes” (pregnant women who have mosquito bed net). None of the outcome variables had missing value; therefore, the whole population was used for the analysis.

### Independent variables

The explanatory variables were selected based on a review of related literature [21–24] and their availability within the data set. The following variables were selected for analysis: maternal age was re-coded (15–24, 25–34, 35–49), marital status (never married, married, cohabitation, widowed), educational level (no education, primary education, secondary education, higher education), ethnicity (Akan, Guan, Dagbani, Grusi), religion (no religion/tradition, Islam, Christianity), health insurance (no, yes), parity (0, 1–3, 4 and above), pregnancy status (no or unsure, yes), frequency of reading newspaper or magazine (not at all, less than once a week, at least once a week), frequency of listening to radio (not at all, less than once a week, at least once a week), frequency of watching television (not at all, less than once a week, at least once a week), owns a mobile telephone (no, yes), use of internet (never used, used internet), head of household sex (male, female), wealth index (poorest, poorer, middle, richer, richest), residence type (urban, rural), distance from health facility (big problem, not a big problem), visited by fieldworker in last 12 months (no, yes), partner’s level of education (no education/don't know, primary, secondary, higher), malaria can be prevented by sleeping under mosquito bed net (no, yes), last 6-month heard/seen malaria messages (no, yes), visited health facility last 12 months (no, yes), and region of residence (Western, Central, Greater Accra, Volta, Eastern, Ashanti, Western North, Ahafo, Bono, Bono East, Oti, Northern, Savannah, North East, Upper East, Upper West) were accounted for. The household wealth variable was calculated using the Principal Component Analysis (PCA) technique. Scores were assigned to households based on their possessions (e.g., television, bicycle, and car) and housing characteristics (e.g., source of drinking water, toilet facilities, and flooring materials) [25].

The national wealth quintiles were created by assigning a household score to each household member, rating them based on their score, and splitting the distribution into five equal groups (each representing 20% of the population) [25]. After checking for missingness, none of our explanatory variables had missing values, so we used the Complete Case Analysis (CCA).

### Data analysis

Data were analyzed using Stata version 14. Both descriptive and inferential statistics were used to analyze data in this study. Descriptive statistics such as frequencies and percentages were used to describe participants' characteristics and to present the prevalence of mosquito bed net use and ownership among pregnant women in Ghana at the univariate level of analysis. Bivariate and multivariate logistic regression analyses were used to assess the factors associated with the ownership and use of mosquito bed nets among pregnant women in Ghana. The bivariate and multivariate regression results were presented as odds ratios (ORs) with their confidence intervals (CI) with statistical significance at *p* value < 0.05. A multicollinearity diagnostic test was performed to determine the variance inflation factors (VIF) for the variables seen to predict mosquito bed use (Min = 1.14, Mean VIF = 1.64, Max = 3.12), and ownership of mosquito bed net (Min = 1.01, Mean VIF = 1.38, Max = 2.20). According to the rule of thumb, none of the variables had a higher VIF than required for exclusion in the multivariate analysis. Taking into account a number of factors, such as statistical significance, the degree of association with the outcome variable, theoretical and practical relevance, and the requirement to account for potential confounders while guaranteeing the absence of multicollinearity, only variables that were not significant during the bivariate analysis were excluded from the final multivariate analysis model. The data used for the analyses were weighted (v005/1000000), and to account for the complex sampling of the survey, the ‘svy’ command in Stata was used for analysis.

## Results

### Participant’s characteristics

Table 1 illustrates the results for the characteristics of the pregnant women. Of the 1111 pregnant women, little more than half (52.9%) were between the ages of 25–34 years and were from the urban setting (51.4%). Majority (60.7%) were married, had secondary education (58.2%), and belonged to the Akan ethnic group (39.2%) with some (16.6%) of the pregnant women from the Ashanti region. Similarly, more than half (78.6%) of the pregnant women were working, 73.0% were Christians and almost all of the pregnant women (96.7%) had health insurance. Of all the participants, 83.6% had no visit from a fieldworker in the last 12 months and most (74.5%) had no problem with the distance to a health facility. The majority (80.1%) of the participants owned a mosquito bed net but slightly more than half (52.4%) had never used the mosquito bed net for sleeping.

**Table 1:** Demographic characteristics of respondents on the use of mosquito bed net

Variables	Categories	Weighted N (%)
Respondent slept under mosquito bed net	No	582 (52.4)
	Yes	529 (47.6)
Have mosquito bed net for sleeping (from household questionnaire)	No	221 (19.9)
	Yes	890 (80.1)
Age	15–24	298 (26.8)
	25–34	588 (52.9)
	35–49	225 (20.3)
Marital status	Never married	102 (9.2)
	Married	674 (60.7)
	Cohabitation	306 (27.5)
	Widowed	29 (2.6)
Educational level	No education	206 (18.6)
	Primary	161 (14.5)
	Secondary	647 (58.2)
	Higher	97 (8.7)
Ethnicity	Akan	435 (39.2)
	Guan	241 (21.6)
	Dagbani	237 (21.3)
	Grusi	198 (17.9)
Employment	Not working	238 (21.4)
	Working	873 (78.6)
Religion	No religion/tradition	38 (3.5)
	Islam	262 (23.6)
	Christianity	811 (73.0)
Health Insurance	No	37 (3.3)
	Yes	1,074 (96.7)
Parity	0	222 (20.0)
	1–3	651 (58.6)
	4 and above	238 (21.4)
Frequency of reading newspaper or magazine	Not at all	1,020 (91.8)
	Less than once a week	67 (6.0)
	At least once a week	24 (2.2)
Frequency of listening to radio	Not at all	414 (37.3)
	Less than once a week	270 (24.3)
	at least once a week	427 (38.4)
Frequency of watching television	Not at all	278 (25.0)
	Less than once a week	120 (10.8)
	At least once a week	713 (64.2)
Owns a mobile telephone	No	184 (16.6)
	Yes	927 (83.4)
Internet Use	Never used	631 (56.8)
	Used internet	480 (43.2)
Sex of household head	Male	790 (71.1)
	Female	321 (28.9)
Wealth index combined	Poorest	218 (19.6)
	Poorer	202 (18.1)
	Middle	208 (18.8)
	Richer	272 (24.5)
	Richest	211 (19.0)
Partner’s Level of Education	No education/don't know	209 (20.9)
	Primary	88 (8.9)
	Secondary	565 (56.6)
	Higher	136 (13.6)
Visited by fieldworker in last 12 months	No	929 (83.6)
	Yes	182 (16.4)
Type of place of residence	Urban	571 (51.4)
	Rural	540 (48.6)
Distance to Health Facility	Big problem	284 (25.5)
	Not a big problem	827 (74.5)
Malaria can be prevention by sleeping under Mosquito bed net	No	441 (40.0)
	Yes	670 (60.0)
Last 6-month heard/seen malaria messages	No	419 (37.8)
	Yes	692 (62.2)
Visited health facility last 12 months	No	260 (23.4)
	Yes	851 (76.6)
Region	Western	73 (6.6)
	Central	114 (10.2)
	Greater Accra	156 (14.0)
	Volta	47 (4.3)
	Eastern	102 (9.1)
	Ashanti	184 (16.6)
	Western North	29 (2.6)
	Ahafo	22 (2.0)
	Bono	41 (3.7)
	Bono East	45 (4.1)
	Oti	36 (3.3)
	Northern	119 (10.8)
	Savannah	34 (3.1)
	North East	33 (3.0)
	Upper East	47 (4.2)
	Upper West	28 (2.5)

### Prevalence of mosquito bed net use and ownership among pregnant women

**Fig. 1:**
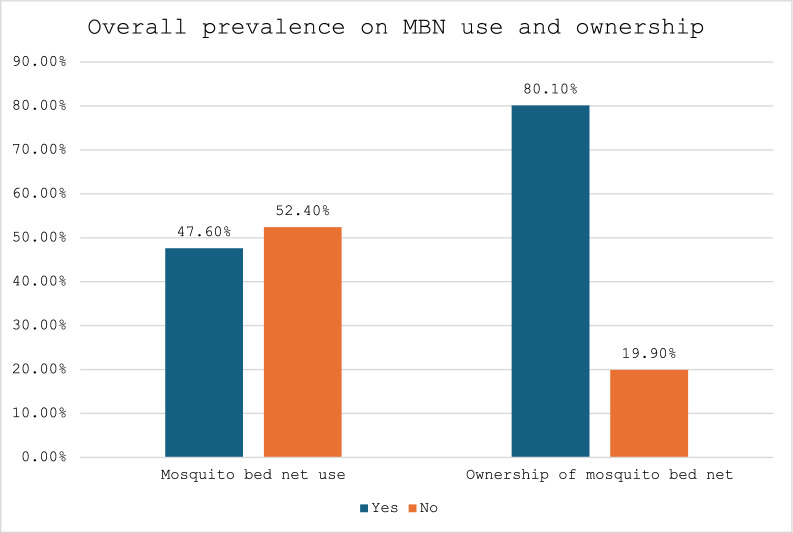
Overall prevalence on mosquito bed net [MBN] use and ownership

**Fig. 2:**
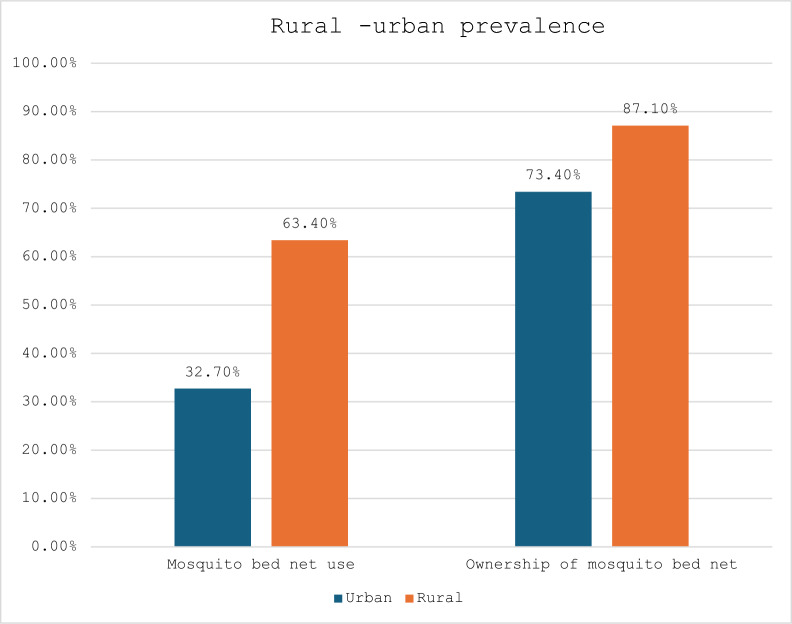
Rural–urban prevalence of mosquito bed net use and ownership

Most of the pregnant women (80.1% [CI 76.8–83.0]) owned mosquito bed nets for sleeping. However, 47.6% [CI 43.9–51.4] of pregnant women used mosquito bed net. Approximately 73.4% [CI 68.1–78.2] of pregnant women who were from urban settings owned mosquito bed net for sleeping. However, 32.7% [CI 28.1–37.8] of them use mosquito bed nets. Likewise, the prevalence of mosquito bed net ownership for sleeping was also higher among pregnant women from rural settings (87.1% [CI 83.7–89.9]), but 63.4% [CI 58.1–68.4] of the pregnant women used the mosquito bed net in rural areas (Figs. 1, 2).

### Factors associated with mosquito bed net use among pregnant women

In a multivariate logistic regression model, parity, wealth index, combined status, partner’s educational level, and region were significantly associated with mosquito bed net use. Parity was found to be significantly associated with the use of the mosquito bed net for sleeping. From the findings, pregnant women with 1–3 children [aOR = 2.07, 95% CI 1.25–3.43] and 4 or more children [aOR = 2.52, 95% CI 1.38–4.59] had higher odds of using mosquito bed net as compared to pregnant women who had no children. In addition, pregnant women who belonged to middle wealth quintiles [aOR = 0.41, 95% CI 0.21–0.82], richer wealth quintiles [aOR = 0.19, 95% CI 0.09–0.41], and richest wealth quintiles [aOR = 0.09, 95% CI 0.03–0.25] had decreased odds of mosquito bed net use as compared to pregnant women from the poorest wealth quintiles. Pregnant women with partners having secondary [aOR = 2.11, 95% CI 1.10–4.06] and higher educational status [aOR = 2.47, 95% CI 1.06–5.74] had a higher odd of using mosquito bed net as compared to those participants with partners having no formal education. Furthermore, pregnant women from the Greater Accra region [aOR = 0.32, 95% CI 0.13–0.78] had lesser odds of mosquito bed net use as compared to their counterparts living in the Western region (Table 2).

**Table 2:** Factors associated with mosquito bed net use among pregnant women

Variables	cOR (95% CI)	aOR (95% CI)
*Educational level*		
No education	Ref	Ref
Primary	0.83 [0.52–1.34]	1.05 [0.54–2.06]
Secondary	0.60** [0.42–0.86]	1.21 [0.62–2.37]
Higher	0.29** [0.14–0.58]	1.23 [0.43–3.55]
*Ethnicity*		
Akan	Ref	Ref
Guan	1.33 [0.84–2.10]	1.28 [0.68–2.40]
Dagbani	1.39 [0.94–2.04]	1.18 [0.58–2.41]
Grusi	2.14** [1.34–3.43]	1.47 [0.75–2.91]
*Religion*		
No religion/tradition	Ref	Ref
Islam	0.25** [0.12–0.55]	0.48 [0.18–1.27]
Christianity	0.25*** [0.12–0.50]	0.58 [0.22–1.50]
*Parity*		
0	Ref	Ref
1–3	1.30 [0.90–1.87]	2.07** [1.25–3.43]
4 and above	2.35*** [1.51–3.65]	2.52** [1.38–4.59]
*Last 6-month heard/seen malaria messages*		
No	Ref	Ref
Yes	0.70*[0.50–0.97]	1.23[0.81–1.87]
*Frequency of watching Television*		
Not at all	Ref	Ref
Less than once a week	0.46** [0.27–0.77]	0.66 [0.35–1.24]
At least once a week	0.29*** [0.20–0.40]	0.82 [0.51–1.33]
*Owns a mobile telephone*		
No	Ref	Ref
Yes	0.54** [0.38–0.77]	1.41 [0.90–2.20]
*Internet*		
Never used	Ref	Ref
Used internet	0.33*** [0.24–0.45]	0.81 [0.50–1.29]
*Wealth index combined*		
Poorest	Ref	Ref
Poorer	1.08 [0.70–1.65]	1.10 [0.66–1.85]
Middle	0.45*** [0.30–0.68]	0.41** [0.21–0.82]
Richer	0.20*** [0.13–0.31]	0.19*** [0.09–0.41]
Richest	0.09*** [0.05–0.16]	0.09*** [0.03–0.25]
*Partner’s educational level*		
No education/don’t know	Ref	Ref
Primary	0.92 [0.50–1.69]	1.38 [0.71–2.67]
Secondary	0.66* [0.46–0.94]	2.11* [1.10–4.06]
Higher	0.34** [0.23–0.67]	2.47* [1.06–5.74]
*Distance to facility*		
Big problem	Ref	Ref
Not a big problem	0.67* [0.48–0.94]	1.01 [0.66–1.56]
*Residence*		
Urban	Ref	Ref
Rural	3.56*** [2.60–4.86]	1.54 [0.99–2.39]
*Region*		
Western	Ref	Ref
Central	1.03 [0.49–2.18]	0.80 [0.32–2.00]
Greater Accra	0.34** [0.15–0.74]	0.32* [0.13–0.78]
Volta	1.46 [0.64–3.31]	0.55 [0.17–1.75]
Eastern	1.01 [0.44–2.31]	0.57 [0.22–1.50]
Ashanti	0.65 [0.32–1.29]	0.55 [0.24–1.27]
Western North	1.33 [0.56–3.20]	0.80 [0.30–2.15]
Ahafo	2.83* [1.29–6.20]	1.27 [0.46–3.48]
Bono	1.42 [0.61–3.32]	1.12 [0.37–3.41]
Bono East	1.92 [0.85–4.36]	1.17 [0.39–3.54]
Oti	2.14 [0.90–5.12]	0.79 [0.26–2.46]
Northern	1.27 [0.61–2.62]	0.47 [0.16–1.36]
Savannah	2.52** [1.28–4.89]	0.83 [0.31–2.26]
North East	1.43 [0.69–2.97]	0.44 [0.16–1.25]
Upper East	2.19 [0.99–4.86]	0.67 [0.22–2.00]
Upper West	1.40 [0.67–2.91]	0.56 [0.18–1.73]

aOR = Adjusted Odds ratio, cOR = Crude Odds Ratio CI = Confidence Interval; *p < 0.05, **p < 0.01, ***p < 0.001; Ref = Reference category

### Factors associated with the ownership of mosquito bed net among pregnant women

Pregnant women whose household heads were females [aOR = 1.73, 95% CI 1.03–2.91] had higher odds of mosquito bed net ownership as compared to pregnant women with male household heads. Again, pregnant women with active health insurance [aOR = 0.29, 95% CI 0.10–0.80] had decreased odds of owning a mosquito net as compared to those pregnant women without health insurance. Pregnant women who resided in rural areas [aOR = 1.97, 95% CI 1.12–3.49] had higher odds of possessing a mosquito bed net than those who stayed in urban settings. Moreover, pregnant women with 1–3 children [aOR = 1.65, 95% CI 1.05–2.58] and 4 or more children [aOR = 2.08, 95% CI 1.03–4.20] had higher odds of owning a mosquito bed net. In addition, pregnant women from the North East region [aOR = 0.27, 95% CI 0.11–0.67] had lesser odds of mosquito bed net ownership as compared to their counterparts living in Western region (Table 3).

**Table 3:** Factors associated with mosquito bed net Ownership among pregnant women

Variables	cOR (95% CI)	aOR (95% CI)
*Health Insurance*		
No	Ref	Ref
Yes	0.24** [0.09–0.62]	0.29* [0.10–0.80]
*Parity*		
0	Ref	Ref
1–3	1.54 [0.99–2.37]	1.65* [1.05–2.58]
4 and above	2.20* [1.14–4.25]	2.08* [1.03–4.20]
*Internet*		
Never used	Ref	Ref
Used internet	0.55* [0.36–0.83]	0.76 [0.42–1.37]
*Household sex*		
Male	Ref	Ref
Female	1.60* [1.04–2.45]	1.73* [1.03–2.91]
*Wealth Index Combined*		
Poorest	Ref	Ref
Poorer	1.32 [0.75–2.33]	1.31 [0.74–2.32]
Middle	0.88 [0.51–1.54]	1.10 [0.53–2.31]
Richer	0.60* [0.37–0.99]	0.89 [0.35–2.25]
Richest	0.49** [0.29–0.82]	0.87 [0.29–2.63]
*Residence*		
Urban	Ref	Ref
Rural	2.44*** [1.67–3.57]	1.97* [1.12–3.49]
*Region*		
Western	Ref	Ref
Central	0.81 [0.32–2.05]	0.88 [0.34–2.26]
Greater Accra	0.39* [0.17–0.92]	0.53 [0.22–1.28]
Volta	0.87 [0.37–2.06]	0.74 [0.30–1.81]
Eastern	2.07 [0.73–5.86]	1.61 [0.55–4.76]
Ashanti	0.67 [0.27–1.64]	0.71 [0.28–1.81]
Western North	0.85 [0.32–2.23]	0.66 [0.24–1.79]
Ahafo	1.47 [0.52–4.20]	1.30 [0.43–3.99]
Bono	1.86 [0.63–5.47]	1.99 [0.62–6.37]
Bono East	1.04 [0.36–2.96]	0.86 [0.28–2.63]
Oti	1.31 [0.48–3.57]	0.99 [0.35–2.81]
Northern	0.53 [0.25–1.12]	0.47 [0.21–1.08]
Savannah	1.11 [0.46–2.66]	0.75 [0.29–1.93]
North East	0.41* [0.18–0.90]	0.27** [0.11–0.67]
Upper East	1.31 [0.47–3.62]	0.96 [0.34–2.73]
Upper West	1.08 [0.99–4.86]	0.84 [0.35–2.03]

aOR = Adjusted Odds ratio, cOR = Crude Odds Ratio CI = Confidence Interval; *p<0.05, **p<0.01, ***p<0.001; Ref = Reference category

## Discussion

One of the cardinal evidence-based malaria prevention measures in Ghana is the utilization of mosquito bed nets among households [3]. The study assessed contributing factors connected with the usage and ownership of mosquito bed nets among pregnant women in Ghana. After controlling for various variables, the following determinants remained significant predictors of the usage of mosquito bed nets, such as parity, region, partner's educational level, and wealth index quintiles. On the other hand, parity, region, household head sex, residence, and health insurance were positively associated with the ownership of mosquito bed nets. Despite recording a higher prevalence rate (80.1%) of ownership of mosquito bed nets among pregnant women in Ghana, the overall prevalence of the use of mosquito bed nets was low (47.6%). This outcome correlates with previous studies that indicated a low proportion of the usage of mosquito bed nets among pregnant women in Ghana (49.2%) [19], Rwanda (57.9%), and East Africa (47.05%) [26]. This could be due to the unbearable heat some of the mosquito bed nets produce in a room that is already warm, making the pregnant women uncomfortable [27, 28]. In addition, the use of mosquito bed nets for other purposes for which they were not intended for such as fishing, fencing gardens, stuffing pillows, covering cargos, screening windows, lack of hanging kits, and enclosing poultry farms [29]. Moreover, the belief of pregnant women that malaria is no longer a serious health issue and the application of various malaria precautionary actions, such as the use of mosquito repellent, among others, may be a reason for the low usage of mosquito bed nets [30]. However, other studies [31, 32] reported a higher prevalence of mosquito bed net utilization. For instance, a study conducted in the Democratic Republic of the Congo and Northern Nigeria found a prevalence of 71.4% [32] and 71.8% utilization rates [31], respectively. A plausible explanation for these variations could be that various countries may have diverse malaria degree of risks as a result of different climatic and geographic conditions, and they also may have different malaria control strategies [1]. For example, the armed conflict crisis in Northern Nigeria has called for the attention of the government to intensify the free distribution of mosquito bed nets in that part of Nigeria to the Internally Displaced Persons (IDPs) to help minimize the burden of malaria [31, 33]. In addition, in the Democratic Republic of Congo, various distribution strategies were adopted from time to time, including a fixed strategy and a door-to-door strategy with hang-up activities, where mosquito bed nets are fixed in the rooms of the individuals [34]. This, therefore, calls for a need to intensify education tailored toward malaria prevention, especially the utilization of mosquito bed nets.

Multiparous pregnant women were more likely to use and own mosquito bed nets than those who were nulliparous. Arguably, one of the places mosquito bed nets are shared is the antenatal care unit, and the more you get pregnant, the more you visit the antenatal care unit, hence possessing mosquito bed nets [1]. In addition, women with multiple pregnancies may have experienced the effects of malaria while pregnant, and so will want to take all precautions to prevent malaria [1]. Furthermore, the antenatal unit also serves as the unit for counseling and education, multiparous women are exposed to varied information on malaria, which helps them to appreciate the use of the mosquito bed net [1].

Consistent with a prior study, pregnant women with spouses with higher education had higher odds of utilizing mosquito bed nets [35]. This may be attributed to the premise that the educated partner may have various knowledge on malaria or might have access to mosquito control educational materials, and is more likely to encourage their wives to utilize mosquito bed nets [26, 35]. Therefore, lower educational levels may lead to a lack of knowledge about the significance of adopting healthy behaviors and how they relate to disease prevention, in this case, the use of mosquito bed nets and how they prevent malaria, as well as how to properly set them up and hang them. By considering the target audience when educating people on the use of mosquito bed nets, this problem may surely be addressed.

Moreover, this study also indicated that women who are pregnant and are from the richest households have lower odds of utilizing mosquito bed nets. This study's finding corroborates prior studies [19, 36, 37] which also reported that those from poorer socioeconomic households had higher odds of utilizing mosquito bed nets. This might be the only defense against mosquito bites for the impoverished. However, wealthy women may live in homes and settings that are well-shielded from mosquitoes, with good drainage systems, or take other precautions to avoid getting bitten [36]. Such techniques may include living in dwellings fortified with mosquito net doors and windows, and the use of mosquito repellent formulas or pesticides that keep insects away from the home, making the use of mosquito bed nets unnecessary [36, 38]. The presence of ceilings, screened doors, and windows provides less entry points for mosquitoes, which may help reduce bites from infectious mosquitoes, hence reducing malaria [2, 39].

Another important finding was that pregnant women who resided in rural areas had higher odds of owning mosquito bed nets, which is in tandem with prior studies [37, 38, 40]. This could mean that malaria prevention campaigns have targeted rural areas considering the fact that these areas are conducive environments for mosquitoes to breed in [36, 41, 42]. In addition, the pregnant women in the village may not be exposed to or cannot afford other means of malaria prevention strategies [22, 38, 40, 43].

Pregnant women who belonged to households headed by females had higher odds of owning mosquito bed nets. A plausible explanation could be that female-headed households frequently prioritize healthcare and preventive measures to keep their families safe from diseases such as malaria [44]. Women may also have more control over their financial and family decisions, allowing them to give preference to health-related purchases such as bed nets. Several malaria prevention initiatives target women as caregivers, providing bed nets through maternal and child health clinics or antenatal care [1]. Female-headed households may have more access to these programs than male-headed households. For instance, during pregnancy, women are routinely given free or subsidized bed nets, increasing the likelihood of ownership in female-led households [1].

However, pregnant women who had active health insurance had lesser odds of owning a mosquito bed net than those without health insurance. Pregnant women with active health insurance may regard themselves as less prone to malaria because of their access to healthcare and treatment [45]. This notion may lessen the need to possess a mosquito bed net. In addition, health insurance may provide pregnant women with a sense of security, leading them to assume they will be able to receive prompt treatment if necessary, lowering their focus on prevention [45].

Relative to the Western region, pregnant women from the Greater Accra and North East regions had decreased odds for the utilization and ownership of mosquito bed nets, respectively. This is supported by a previous study in Sierra Leone [23]. This could be due to regional differences in climate, topography, and environmental conditions that breed mosquitoes [16, 46, 47]. In addition, those in cities like Greater Accra might have adopted other methods for preventing malaria and are less likely to use mosquito bed nets. The disparities in access may result from regional variations in mosquito bed net distribution programs [23]. Fewer mosquito bed nets may be available in areas with weaker distribution networks, and economic differences in the various regions may also contribute to low access to mosquito bed nets in the North East**.** North East may also face limited health infrastructures, which may decrease distribution points and lower income levels, which may decrease their accessibility to the mosquito bed nets. This finding is contrary to prior studies [35], which revealed a higher likelihood of pregnant women in the capital cities of Rwanda using mosquito bed nets. This could be due to the high endemicity of malaria in those cities and constant accessibility to free mosquito bed net distribution and education on the importance of mosquito bed net use. In addition, the trust instilled in these pregnant women by the healthcare providers encourages the pregnant women in the cities of Rwanda to continuously use the mosquito bed net.

### Limitations of the study

This research used the recent nationally representative data: the 2022 Demographic and Health Survey. The survey's representativeness, dual phase sampling approach, and analytical rigor all contribute to the validity of the results. However, the study was cross-sectional in nature and, therefore, may not determine causal correlations between factors of mosquito bed net ownership and use. The use of mosquito bed nets was assessed the night before the survey; therefore, it may not accurately reflect usage changes over time. Recall and social desirability biases may have influenced the self-reported findings. The data did not account for the seasonality of mosquito abundance, which could impact mosquito bed net ownership and utilization. However, we feel this did not significantly impair the accuracy of the results obtained. The study's use of secondary data did not account for potential neighborhood and national factors influencing pregnant women's mosquito bed net use and ownership.

## Conclusion

The study revealed a high rate of mosquito bed net ownership among pregnant women. However, pregnant women were unable to properly use the mosquito bed net, because the prevalence of mosquito bed net use among pregnant women was low. The study also found that having a household head, health insurance, type of residence and parity all had a significant impact on mosquito bed net ownership among pregnant women in Ghana. On the other hand, parity, household wealth, partner's level of education, and region all had an impact on mosquito bed net utilization among pregnant women. However, considering the relatively low proportion of mosquito bed net utilization among pregnant women, coupled with the fact that pregnant women are especially susceptible to malaria, there is a need for public health practitioners and clinicians to develop an awareness and educational interventions tailored toward improving mosquito bed net use among pregnant women.

## Data Availability

The dataset is publicly available at the Measure DHS repository (https://dhsprogram.com/data/dataset/Ghana_Standard-DHS_2022.cfm?flag=1).

## Abbreviations

aOR: Adjusted odds ratio
CI: Confidence interval
cOR: Crude odds ratio
GSS: Ghana statistical service
ICF: International coaching federation
IPTp: Intermittent preventive treatment of malaria in pregnancy
KOICA: Korean International Cooperation Agency
LMICs: Lower- and middle-income countries
MIP: Malaria in pregnancy
NMCP: National Malaria Control Programme
PCA: Principal component analysis
PMI: President's malaria initiative
PPS: Probability proportional to size
SSA: Sub-Saharan Africa
UK AID: United Kingdom Agency for International Development
UNFPA: United Nations Population Fund
UNICEF: United Nations International Children’s Fund
USAID: United States Agency for International Development
VIF: Variance inflation factors
WHO: World Health Organization
